# Beginning A Patient-Centered Approach in the Design of A Diabetes Prevention Program

**DOI:** 10.3390/ijerph110202003

**Published:** 2014-02-14

**Authors:** Richard W. Seidel, Kimberlee A. Pardo, Paul A. Estabrooks, Sarah S. Wall, Brenda M. Davy, Fabio A. Almeida

**Affiliations:** 1 Department of Psychiatry, Virginia Tech Carilion School of Medicine, 2017 South Jefferson Street, Roanoke, VA 24014, USA; 2 Fralin Translational Obesity Research Center, Department of Human Nutrition, Foods and Exercise, Virginia Tech, 215 War Memorial Hall, Blacksburg, VA 24061, USA; E-Mails: skim13@vt.edu (K.A.P.); estabrkp@vt.edu (P.A.E.); wenyou@vt.edu (W.Y.); sarahsw@vt.edu (S.S.W.); bdavy@vt.edu (B.M.D.); falmeida@vt.edu (F.A.A.); 3 Department of Family and Community Medicine, Carilion Clinic, 101 Elm Avenue, Roanoke, VA 24013, USA; E-Mail: paestabrooks@carilionclinic.org; 4 Department of Agriculture and Applied Economics, Virginia Tech Carilion School of Medicine, 321A Hutcheson Hall, Blacksburg, VA 24061, USA

**Keywords:** diabetes prevention program, technology-enhanced intervention, low income populations, patient preferences

## Abstract

*Objective:* The purpose of this study was to identify patient preferences for different components of a local diabetes prevention program that would improve reach.A secondary purpose was to determine if patient characteristics were related to program preferences. *Methods:* Participants were identified through electronic medical records from two family medicine clinics in Virginia. Participants completed a mailed survey addressing demographics, economic status, risk factors for diabetes, and preferences regarding diabetes prevention interventions—delivery mode, program length, and duration. *Results:* Twenty-nine percent of eligible participants responded (n = 142); 83% of participants were at risk for diabetes and 82% had a household income <$20,000.When presented with the choice between a class-based *vs.* a technology-based program, 83% preferred a technology-based program. Whites were less likely to choose the technology-based program, with no significant differences based on age, education, income, or gender. *Conclusions:* Contrary to beliefs that lower income individuals may not use technology-based interventions, lower socioeconomic patients indicated a preference for a technology- and telephone-supported diabetes prevention program over in-person class approaches. Findings provide formative data to support the design of a patient-centered, technology-enhanced diabetes prevention program in a real-world setting, thereby increasing potential participation and reach.

## 1. Introduction

With the ever quickening pace of life in modern societies, individuals are bombarded with information and choices. Additionally, given that human beings seek out pleasure, avoid pain, and are designed to do these things as efficiently as possible [1], it is imperative that when designing health behavior change interventions that they are as aligned as possible with the preferences of the participants.

This effort is particularly important because the predicted trends in the prevalence of type 2 diabetes (T2DM) pose an enormous public health concern. Approximately 8% of USA adults are currently diagnosed with T2DM with an additional 5% having undiagnosed T2DM. Estimates indicate that by 2050 one in three adults will have T2DM [2]. People at greatest risk for diabetes are those who are overweight and or obese, and have higher than normal blood glucose concentrations, or prediabetes [3,4]. Consequently, these individuals are also at higher risk for cardiovascular disease [5], and may spend 42% more in annual medical costs than their healthy-weight counterparts [6].

However, prediabetes and T2DM do not equally affect geographies and populations across the USA [7]. In Virginia, the prevalence is greater among African Americans (13.5%), persons with incomes below $24,000 (15.4%), and those with less than a high school education (15.0%) [7]. This disproportionate burden of diabetes on low socioeconomic status (SES) populations highlights the need to develop cost-effective, wide-reaching diabetes prevention interventions [8,9].

### 1.1. Generalizability of Diabetes Prevention Programs

The Diabetes Prevention Program (DPP) demonstrated that modest weight loss achieved through diet and exercise was superior to medication in delaying the onset of T2DM [10]. The DPP found that 30 min of physical activity (PA) per day 5 times a week, coupled with a 5%–10% weight loss, resulted in a 58% reduction in the incidence of diabetes [10].

Subsequently, many researchers have endeavored to translate the DPP into community and clinical settings [11,12,13,14,15,16,17,18,19,20,21,22], yet establishing wide reaching DPP-based interventions in clinical settings remains a challenge, and prevalence rates continue to rise [2,7,23].

Laws and colleagues recently published a review of DPP interventions and concluded that the lack of reporting on external validity dimensions has significantly limited the ability to generalize findings [24] Furthermore, less than 20% of studies they reviewed described how representative subjects were of the target population, and few reported on key components of reach , such as the associations between SES , morbidity and mortality, prevention and risk factors, and access to proper health care [23].

### 1.2. Interactive Technology and Diabetes Prevention Programs

Diabetes prevention programs face several challenges including providing individual participants with the right mixture of intensity, personalization, and flexibility to maximize effect; reaching those at greatest risk; and delivering effective interventions in a sustainable format [25]. Intensive interventions are often expensive and offer little flexibility in the timing and availability of sessions, often resulting in low participation rates among those who could benefit the most [25]. This generally makes program adoption and sustainability within healthcare settings unfeasible. Interactive technologies may offer alternative avenues for the delivery of intervention content to high-risk individuals at convenient times and locations, thus providing possible solutions to DPP challenges [25,26,27,28,29,30,31,32,33]. However, limited data exists on patient-centered approaches to determine the utility of these intervention delivery channels for T2DM prevention. Further, much remains to be learned about what interventions are most appealing to which people.Therefore, the purpose of this study was to identify patient preferences for different components of a proposed local diabetes prevention program that would improve the reach of those at greatest need (low SES and minorities) and be sustainable in local healthcare clinics. A secondary purpose was to determine if the preferences for different program components was related to patient characteristics.

## 2. Methods

### 2.1. Study Setting and Population

Patients at risk for developing T2DM were identified through electronic medical records (EMR) of two family medicine clinics serving the Roanoke, VA area. A total of 490 patients were identified using the following ICD-9 diagnoses: prediabetes; glucose intolerance; hyperinsulinism; metabolic syndrome; obesity, morbid; simple Obesity; and/or abnormal weight gain [34].Identified patients received a letter from their physician with a survey, a $2.00 cash gift, and a return envelope. Approximately 10 days after the mailing, potential participants were called to follow up. This study and procedures were approved by the Carilion Clinic Institutional Review Board.

### 2.2. Key Outcome Measures

Participants were asked about their willingness to participate in two technology-based programs: (a) an educational DVD followed by automated interactive voice response (IVR) telephone calls, or (b) an in-person small group session followed by IVR calls. Additionally, participants were asked their preference for program length, the frequency and duration of IVR phone calls. The survey also included items from the diabetes risk assessment, which included height, weight, race, ethnicity, family diabetes history, and physical activity behaviors [35]. Analyses were conducted using SPSS statistical software, version 21.0. Simple descriptive and frequency statistics were used to characterize the sample. Chi-square tests were used to evaluate the representativeness of study participants when compared to the surrounding communities served by both clinics and the larger Roanoke population. Logistic regression was used to investigate differences in willingness to participate and program of choice adjusting for standard demographic variables. Finally, chi-square analyses evaluated differences in program length and duration of calls. All significance levels were set at *p* < 0.05.

## 3. Results

One hundred and forty two surveys, 29% of those mailed, were returned. The average age of respondents was 52.7, with 77% female, 43% African-American, and 44% earning $10,000 or less over the last year (Table 1).

**Table 1:** Representativeness of participants compared to Roanoke, Virginia.

Characteristic	Respondents (*n* = 142)	Census Tract^ c^ (*n* = 8,435)	Roanoke, VA (*n* = 75,278)
**Age (mean)**	52.7	41.3	38.7
**Female (%)**	77.0***	54.5	53.3
**BMI (kg/m^2^)**	39.7	n/a	n/a
**Obese (%)**	92.6	n/a	34
**High Risk for Diabetes (%)^a^**	83.1	n/a	n/a
**Race (%)**			
White	53.7	63.1***	54.0
Black/African-American	42.5***	34.4	35.0
American-Indian/Alaskan Native	1.5	0.5	0.5
Other	2.3	2.1	10.5***
**Education (%)**			
Less than high school graduate	25.2	22.8	11.7***
High school graduate or higher no Bachelors’ degree	66.6	64.3	52.2***
Bachelors’ degree or higher	8.2	13.0	36.1***
**Employment Status (%)**			
Full-time or part-time	23.0***	56.0	56.2
Unemployed	17.0	5.2***	23.7
Other (retired, homemaker)	22.2	26.8	12.9***
On Disability (SSI)	37.8***	12.0	7.2
**Current Tobacco Smokers (%)**	26.6	n/a	26.0
**Annual Income < $20,000 (%)^b^**	67.7	27.7 ^b^	32.6 ^b^
**Health Insurance Status (%)**			
Uninsured	26.2	20.7	24.3
**Access to Telephone (%)**	98.6	n/a	n/a
**Access to DVD Player (%)**	90.0	n/a	n/a

Notes: ^a ^[36] Heikes, K.E.; Eddy, D.M.; Arondekar, B.; Schlessinger, L. Diabetes Risk Calculator: a simple tool for detecting undiagnosed diabetes and pre-diabetes. Diabetes care. 2008; 31:1040–1045; ^b^ This value is not on the same threshold. 32.6% of people in the City of Roanoke earn <$25,000; ^c^ Combined census tract data from the American Community Survey 2012 [37]; * < 0.05; ** < 0.01; *** < 0.001.

The average BMI was 39.7and 83% of respondents were at high risk for developing T2DM (*i.e.*, scored ≥ 5 on diabetes risk assessment [35]). Compared to the greater Roanoke area, study participants were more likely to be female (77.0% *vs.* 53.3%), black (42.5% *vs.* 35.0%), and less educated (Bachelors’ degree or higher, 8.1% *vs.* 36.1%) (Table 1). Furthermore, when compared to the census tract data from the surrounding communities served by both clinics, study participants were more likely to be female and black, while there were no differences in educational level (Table 1).

Eighty-five percent of respondents were willing to participate in either of the two programs.Of those, over 75% indicated preference for weekly calls for the first 2–3 months, then bi-weekly calls for months 4–6, and then monthly calls through 12 months. Chi-Square tests revealed that male respondents (55% *vs.* 31% 12+months, *x*^2^(5) = 14.363, *p* < 0.01) and those with no health insurance (28% *vs.* 10% 18+ months, *x*^2^(4) = 9.721, *p* < 0.05) thought the frequency of calls should last longer. There were no differences according to disability status, education, income, or race. Finally, 85% of respondents indicated that call duration should be less than 10 minutes with no significant differences according to sex, insurance status, disability status, education, income, or race.

Additionally, logistic regression results showed that older respondents (OR = 0.91; CI = 0.86–0.97) and those with a higher number of children living in the household (OR = 0.73; CI = 0.56–0.94) were less likely to be interested in participating. There were no differences in interest based on education, income, race, and sex (Table 2). 

**Table 2:** Logistic regression results for willingness to participate.

Characteristic	OR	SE	CI (95%)
Age	0.91**	0.03	0.86–0.97
Female	1.20	1.04	0.22–6.56
White	1.73	1.12	0.48–6.15
Health Insured	0.43	0.40	0.07–2.57
Smoker	0.32	0.25	0.07–1.46
Education	1.18	0.22	0.82–1.69
Income	1.08	0.28	0.65–1.79
BMI	1.02	0.04	0.95–1.11
Number of Children	0.73*	0.09	0.56–0.94
Number of People in Household	1.07	0.22	0.71–1.60

Notes: Wald Chi^2^(10) = 18.81, *p* < 0.05; SE = Standard Error; OR = Odds Ratio (the OR here is OR adjusting for the demographic variables shown in the table); CI = Confidence Interval; *p< 0.05, **p <0.01.

Of those that indicated a willingness to participate in either program, 83% preferred the DVD-based over the small group-based version. Logistic regression results demonstrated that whites were significantly less likely to choose the DVD option (OR = 0.23; CI = 0.06–0.81). There were no differences regarding age, education, income, and sex (Table 3).

**Table 3:** Logistic regression results for DVD-based program preference.

Characteristic	OR	SE	CI (95%)
Age	1.02	0.03	0.96–1.09
Female	1.47	1.12	0.33–6.56
White	0.23*	0.15	0.06–0.81
Education	1.07	0.21	0.72–1.59
Income	1.02	0.18	0.72–1.43

Notes: Wald Chi^2^(5) = 10.22, *p* < 0.1; SE = Standard Error; OR = Odds Ratio (the OR here is OR adjusting for the demographic variables shown in the table); CI = Confidence Interval; *p < 0.05.

## 4. Discussion

This project sought to determine participants’ preferences for technology-based diabetes prevention programs, and how demographic characteristics may be related to those choices. Current literature and growing epidemiological data has suggested that lower SES individuals are less likely to participate in such programs [38,39]. Technology-based programs nevertheless continue to show promise [26,40,41,42,43,44,45], and our study indicated that a large proportion (85%) of respondents were amenable to technology-based programs to prevent diabetes. More importantly, there were no associations between willingness to participate and SES status (*i.e.*, income, education), suggesting that members of lower SES groups, who suffer from a disproportionate burden associated with T2DM, would be just as likely to participate in technology-based programs. Our study sample included a higher proportion of lower SES individuals indicating that technology-based programs could be a promising avenue for reaching this population.

Most respondents willing to participate in a future program preferred a DVD and IVR-based program. Furthermore, there were no differences in program preference based on SES, which may not be surprising since the vast majority of participants had access to a telephone (98.6%) and a DVD player (90%). A large majority preferred a program lasting at least 12 months, with ongoing follow-up throughout the year. These results suggest patient preferences are consistent with the literature that recommends that programs last at least one year [33].

### Limitations

The cross-sectional nature of the data does not allow for causal inferences to be made or whether these interventions would be effective. Further, the survey responses indicate a potential interest in future programs, but not actual participation in such programs. Nonetheless, intention to engage in a particular behavior (*i.e.*, participate in a program) has been shown to be predictors of future behavior (*i.e.*, participation) [46]. Additionally, the study included patients from only two clinics; therefore, the results may not generalize to a broader population or even beyond the 29% of patients that responded. Finally, our modest response rate could indicate that only those who are most motivated responded to the mailings. Nevertheless, our findings are of particular note because of the substantial proportion of participants who come from SES categories that are traditionally less likely to participate in surveys and scientific studies [47].

## 5. Conclusions

The findings from this investigation provide formative data to support the development and implementation of a patient-centered, technology-enhanced, diabetes prevention program within areal-world primary health care setting with the goal to increase the reach of such interventions.In particular, the high proportion of lower SES respondents provides important information regarding program characteristics that may be more attractive to them. In fact, potential participants appear to prefer brief follow-up phone calls, using a schedule that thins over the course of the program.The willingness of individuals from lower SES status, who are at high risk for developing T2DM to participate in technology-based programs also indicate that these strategies may be a viable alternative to traditional in-person diabetes prevention efforts. Finally, these interventions could be delivered at a lower cost using fewer clinical resources, thereby reducing the financial burden on both individuals and the health care system.
